# Cyclopamine tartrate, an inhibitor of Hedgehog signaling, strongly interferes with mitochondrial function and suppresses aerobic respiration in lung cancer cells

**DOI:** 10.1186/s12885-016-2200-x

**Published:** 2016-02-24

**Authors:** Md Maksudul Alam, Sagar Sohoni, Sarada Preeta Kalainayakan, Massoud Garrossian, Li Zhang

**Affiliations:** Department of Molecular and Cell Biology, Center for Systems Biology, University of Texas at Dallas, Mail Stop RL11, 800 W. Campbell Road, Richardson, TX 75080 USA; Logan Natural Products, 2528 Royal Troon Dr, Plano, TX 75025 USA; The Cecil H. and Ida Green Distinguished Chair, Department of Biological Sciences, The University of Texas at Dallas, Mail Stop RL11, 800 W. Campbell Road, Richardson, TX 75080 USA

**Keywords:** Non-small-cell lung cancer (NSCLC), Hedgehog signaling, Cyclopamine tartrate, SANT1, Oxygen consumption, Mitochondrial fragmentation, ROS, Apoptosis, Glutamine depletion

## Abstract

**Background:**

Aberrant Hedgehog (Hh) signaling is associated with the development of many cancers including prostate cancer, gastrointestinal cancer, lung cancer, pancreatic cancer, ovarian cancer, and basal cell carcinoma. The Hh signaling pathway has been one of the most intensely investigated targets for cancer therapy, and a number of compounds inhibiting Hh signaling are being tested clinically for treating many cancers. Lung cancer causes more deaths than the next three most common cancers (colon, breast, and prostate) combined. Cyclopamine was the first compound found to inhibit Hh signaling and has been invaluable for understanding the function of Hh signaling in development and cancer. To find novel strategies for combating lung cancer, we decided to characterize the effect of cyclopamine tartrate (CycT), an improved analogue of cyclopamine, on lung cancer cells and its mechanism of action.

**Methods:**

The effect of CycT on oxygen consumption and proliferation of non-small-cell lung cancer (NSCLC) cell lines was quantified by using an Oxygraph system and live cell counting, respectively. Apoptosis was detected by using Annexin V and Propidium Iodide staining. CycT’s impact on ROS generation, mitochondrial membrane potential, and mitochondrial morphology in NSCLC cells was monitored by using fluorometry and fluorescent microscopy. Western blotting and fluorescent microscopy were used to detect the levels and localization of Hh signaling targets, mitochondrial fission protein Drp1, and heme-related proteins in various NSCLC cells.

**Results:**

Our findings identified a novel function of CycT, as well as another Hh inhibitor SANT1, to disrupt mitochondrial function and aerobic respiration. Our results showed that CycT, like glutamine depletion, caused a substantial decrease in oxygen consumption in a number of NSCLC cell lines, suppressed NSCLC cell proliferation, and induced apoptosis. Further, we found that CycT increased ROS generation, mitochondrial membrane hyperpolarization, and mitochondrial fragmentation, thereby disrupting mitochondrial function in NSCLC cells.

**Conclusions:**

Together, our work demonstrates that CycT, and likely other Hh signaling inhibitors, can interrupt NSCLC cell function by promoting mitochondrial fission and fragmentation, mitochondrial membrane hyperpolarization, and ROS generation, thereby diminishing mitochondrial respiration, suppressing cell proliferation, and causing apoptosis. Our work provides novel mechanistic insights into the action of Hh inhibitors in cancer cells.

**Electronic supplementary material:**

The online version of this article (doi:10.1186/s12885-016-2200-x) contains supplementary material, which is available to authorized users.

## Background

Hedgehog (Hh) signaling is a key regulator of development and stem cell fate in animals [1]. Dysregulation of the Hh pathway is responsible for various developmental malformations, such as holoprosencephaly [2]. Aberrant activation of the Hh signaling is implicated in a variety of cancers, such as prostate cancer, gastrointestinal cancer, lung cancer, pancreatic cancer, ovarian cancer, and basal cell carcinoma [3–8]. Therefore, Hh signaling pathway has become a therapeutic target for treating many types of cancers. Particularly, a great deal of efforts have been focused on targeting smoothened (SMO), a G protein-coupled receptor mediating Hedgehog (Hh) signaling [9]. Many SMO inhibitors have been generated and tested, and all have shown efficacy as anti-tumor agents [10]. For example, vismodegib is the first FDA-approved SMO inhibitor for the treatment of advanced and metastatic basal cell carcinoma. Currently, vismodegib and many other SMO inhibitors are being investigated in clinical trials in a range of advanced cancers [9, 10]. Consequently, understanding the molecular actions of such inhibitors can be of great value to the improvement of therapeutic strategies for many types of cancers.

The first identified inhibitor of Hh signaling was cyclopamine, a molecule isolated from corn lilies [11, 12]. Cyclopamine binds to and inhibits SMO. Cyclopamine has been very valuable for understanding the function of Hh signaling and has been widely used as an Hh inhibitor in cell and murine models of various tumors [3–5, 13–15]. However, the usefulness of cyclopamine (Cyc) as a therapeutic drug is hindered by its poor aqueous solubility [16]. To improve the solubility and efficacy of Cyc, Dr. Garrossian, a contributor of this manuscript, generated cyclopamine tartrate (CycT) [17]. Indeed, CycT is water soluble, and its activity in inhibiting Hh signaling is higher than Cyc. Furthermore, CycT is effective in causing tumor shrinkage in two mouse models of basal cell carcinomas [17]. Therefore, we decided to examine the efficacy of CycT in inhibiting the proliferation and function of an array of non-small-cell lung cancer (NSCLC) cell lines.

Previously, using a matched pair of cell lines representing normal nonmalignant and NSCLC cells developed from the same patient, we found that oxygen consumption is intensified in NSCLC cells and tumors [18]. Specifically, the rates of both glucose and oxygen consumption in NSCLC HCC4017 cells are elevated, with the elevation of oxygen consumption greater than that of glucose consumption. Inhibition of mitochondrial respiration interferes strongly with NSCLC cell function, proliferation and migration [18]. In this paper, we sought ways to suppress aerobic respiration, which may lead to novel therapeutic strategies to treat lung cancer and other cancers as well. Firstly, we found that oxygen consumption is intensified in an array of NSCLC cell lines. Secondly, we demonstrated that CycT and another SMO inhibitor SANT1, like glutamine depletion, suppress the rates of oxygen consumption and the rates of cancer cell proliferation. Further analyses of various cellular functions showed that CycT, as well as SANT1, increases ROS generation and mitochondrial membrane potential in NSCLC cells. Ultimately, these SMO inhibitors cause mitochondrial fragmentation, leading to apoptosis. Our studies uncovered a novel mode of anticancer action of an Hh inhibitor, which may have broad implications in the development and application of many Hh inhibitors currently being tested.

## Results

### Cyclopamine tartrate (CycT), like glutamine depletion, strongly suppresses oxygen consumption in NSCLC cells

**Fig. 1:**
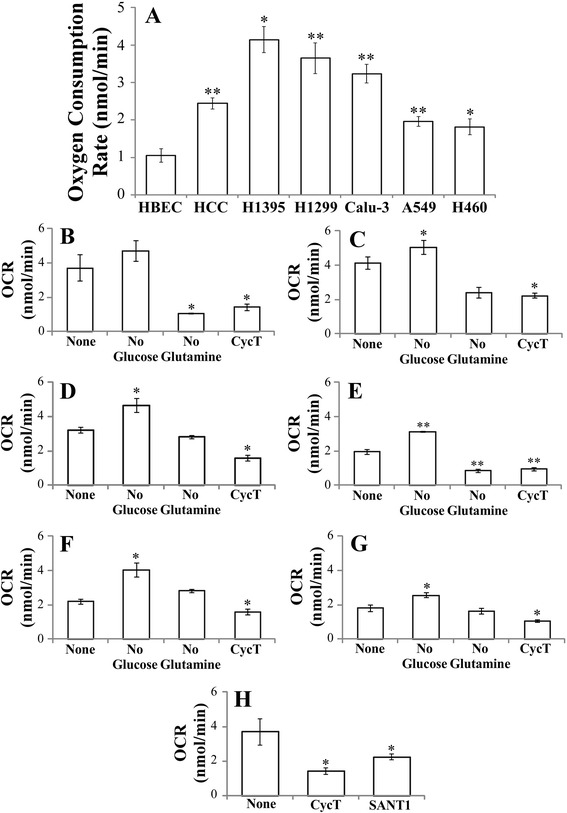
**a** The rates of oxygen consumption are intensified in various NSCLC cell lines. **b-g** CycT, like glutamine depletion, strongly diminished oxygen consumption rates in NSCLC H1299 (**b**), H1395 (**c**), Calu-3 (**d**), A549 (**e**), HCC4017 (**f**), and H460 (**g**) cells. NSCLC cell lines were cultured in their normal medium or in medium lacking glucose or glutamine, or treated with CycT, as indicated. **h** SMO inhibitor SANT1, like CycT, can diminish oxygen consumption in NSCLC cells. H1299 cells were treated with CycT or SANT1. The rates of oxygen consumption were measured. The data shown were averages of at least three independent measurements. For statistical analysis, the values were compared to that in nontumorigenic HBEC lung cells (**a**) or those in normal culture medium (in B-G), by using Welch 2-sample *t*-test. *, *p* value < 0.05; **, *p* value < 0.005

Previously, we showed that the rate of oxygen consumption in NSCLC HCC4017 cells is intensified compared to the nonmalignant HBEC cells representing normal lung epithelial cells from the same patient [18], as shown in Fig. 1a. Additionally, we measured oxygen consumption rates in five other NSCLC cell lines and found that they were substantially increased in all NSCLC cell lines (see Fig. 1a). Furthermore, we examined the effect of glucose depletion, glutamine depletion, and CycT on the rates of oxygen consumption in these NSCLC cell lines. We found that glucose depletion generally enhances oxygen consumption rates, while glutamine depletion diminishes the rates (see Fig. 1b-g). Glucose and glutamine are two critical fuels for cancer cells [19, 20]. When glucose is limiting, the cells use glutamine, and vice versa. Thus, these results showed that in the absence of glucose, glutamine supports intensified oxygen consumption in cancer cells. In the absence of glutamine, even in the presence of glucose, oxygen consumption was substantially reduced (Fig. 1b-g). Notably, CycT diminished the rates of oxygen consumption in cancer cells largely to the same extent as glutamine depletion. Likewise, another SMO inhibitor SANT1 [21, 22] diminished oxygen consumption in NSCLC cells, as expected. These results indicate that CycT can cause the same effect as glutamine depletion on cancer cell metabolism and aerobic respiration.

### CycT causes apoptosis in NSCLC cells

**Fig. 2:**
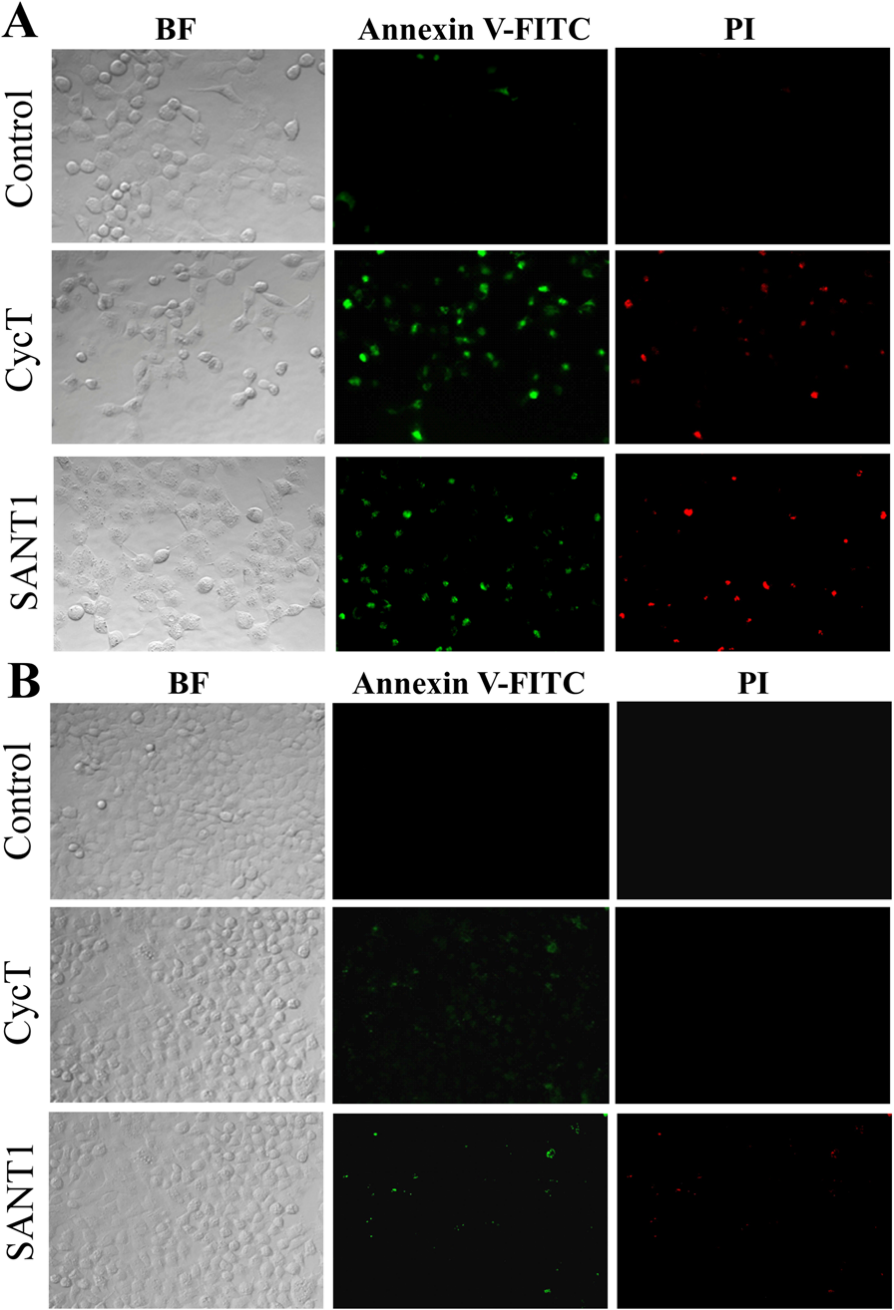
CycT and SANT1 induce apoptosis in H1299 (**a**) and A549 (**b**) NSCLC cell lines. The NSCLC cells were treated with CycT or SANT1 for 24 h. Then cells were subjected to apoptosis assay by using Annexin V-FITC and Propidium Iodide (PI) staining. The images of cells were captured with bright field microscopy (BF) or with fluorescent microscopy with a FITC or rhodamine (for PI) filter

The data shown above revealed a strong effect of CycT on aerobic respiration. Thus, we further examined the effect of CycT on NSCLC cell proliferation. We found that CycT diminishes the proliferation and survival of NSCLC cells, although the sensitivity of different cell lines to CycT varies (see Additional file 1: Fig. S1). We also tested whether CycT causes apoptosis in NSCLC cells by using Annexin V and propidium iodide (PI) staining. We found that CycT indeed causes apoptosis in NSCLC cells, albeit with varying efficacy in different NSCLC cell lines. For example, after 24 h of treatment with CycT, H1299 cells were mostly apoptotic, as detected by Annexin V staining (Fig. 2a). PI staining further showed that a fraction of these apoptotic H1299 cells were in the late apoptotic stage. A549 cells, as shown by the proliferation rates in Additional file 1: Fig. S1, were more resistant to CycT (see Fig. 2b). After 24 h of treatment, only a fraction of the cells showed signs of apoptosis, as detected by Annexin V staining. No A549 cells were in late apoptotic stage (see Fig. 2b). Nonetheless, our results showed that CycT can cause apoptosis in NSCLC cells. Notably, another SMO inhibitor SANT1, like CycT, also exerted similar effects on NSCLC cells (Fig. 2a and b).

### CycT does not exert a considerable effect on heme metabolism

**Fig. 3:**
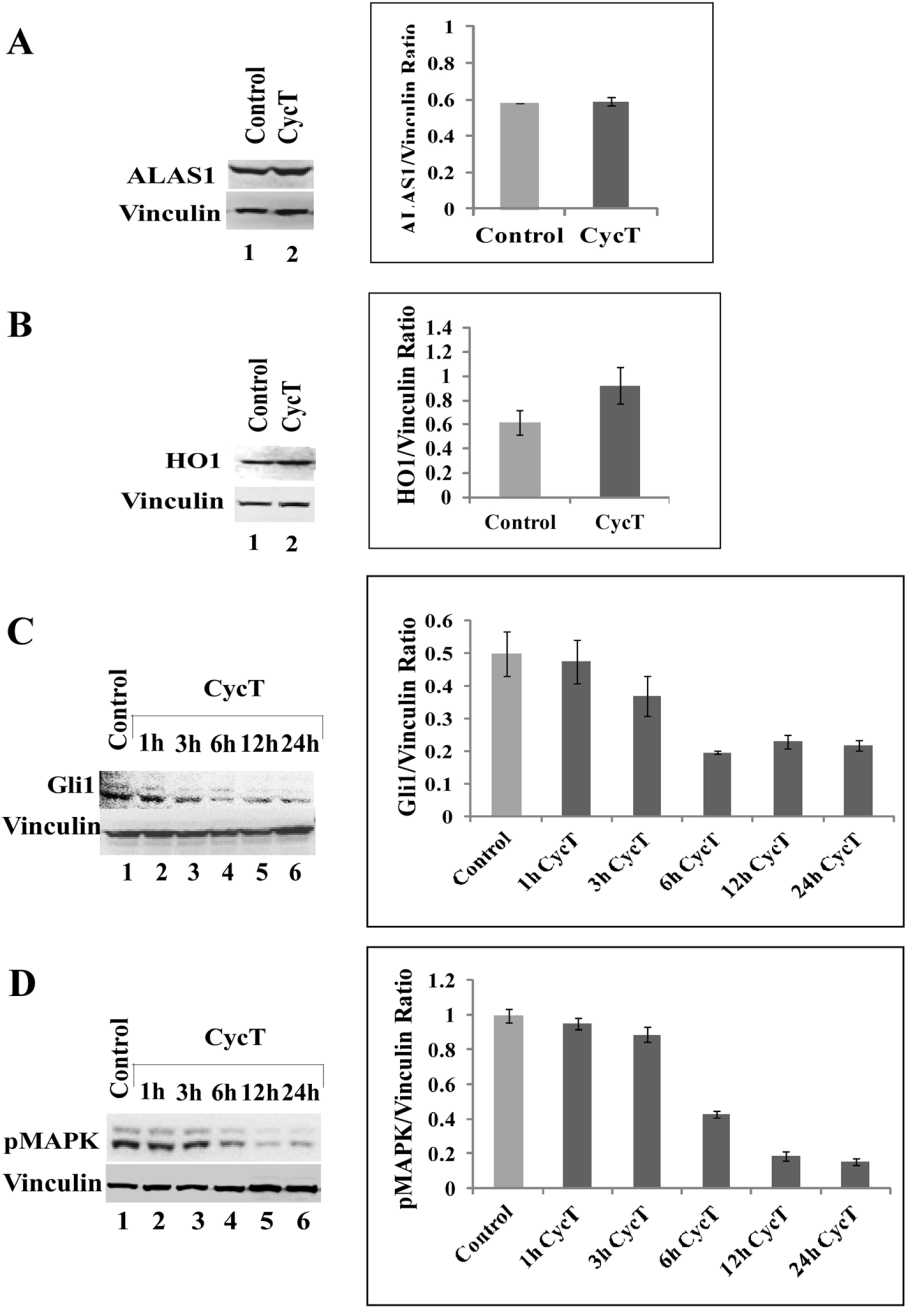
The effect of CycT treatment on the levels of ALAS1 (**a**), HO1 (**b**), Gli1 (**c**), and phospho-p44/p42 MAPK (**d**). The NSCLC A549 cells were cultured and treated with CycT for 24 h [lane 2 in (**a**) and (**b**)] or without CycT [lane 1 in (**a**) and (**b**)]. In (**c**) and (**d**), cells were treated without (lane 1) or with CycT for 1 (lane 2), 3 (lane 3), 6 (lane 4), 12 (lane 5), and 24 (lane 6) hours as indicated. Protein extracts were prepared and the levels of the proteins were detected by Western blotting. The protein level of vinculin was used for normalization. For statistical analysis, the levels in treated cells were compared to the levels in untreated cells, by using Welch 2-sample *t*-test. *, *p* value < 0.05

Heme is a central factor in aerobic respiration and oxidative phosphorylation [23]. Previously, we have shown that limiting intracellular heme levels strongly diminishes mitochondrial respiration and NSCLC cell proliferation and migration [18]. Therefore, we examined whether CycT impacts heme synthesis and metabolism. We found that CycT does not significantly affect the rate of heme synthesis in NSCLC cells (data not shown). Likewise, we found that CycT does not significantly affect the protein levels of the rate-limiting heme synthetic enzyme ALAS1 and the degradation enzyme HO1 (see Fig. 3a and b). For a control, we showed that CycT reduces the level of the Hh signaling target Gli1 (Fig. 3c), as expected. Furthermore, we found that CycT treatment reduced the levels of phosphorylated p44/42 MAPK. The activation of p44/42 MAPK signaling pathway has been shown to be critical for Hh signaling previously [24]. These results show that CycT does not affect aerobic respiration by impacting heme metabolism.

### CycT increases ROS generation and interferes with mitochondrial function in NSCLC cells

**Fig. 4:**
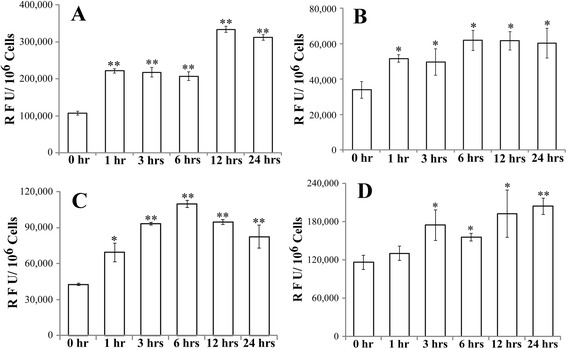
CycT treatment increases ROS production in NSCLC H1299 (**a**), A549 (**b**), and H460 (**c**) cells. **d** SANT1 treatment also increases ROS production in NSCLC H1299 cells. NSCLC cells were treated with CycT or SANT1 for the indicated time periods. Then cells were incubated with 2,7-dichlorodihydrofluorescein diacetate (DCFH-DA) for 30 min. Fluorescence intensity was measured and normalized according to cell density. For statistical analysis, the levels in CycT-treated cells were compared to the levels in untreated cells, by using Welch 2-sample *t*-test. *, *p* value < 0.05; **, *p* value < 0.005

**Fig. 5:**
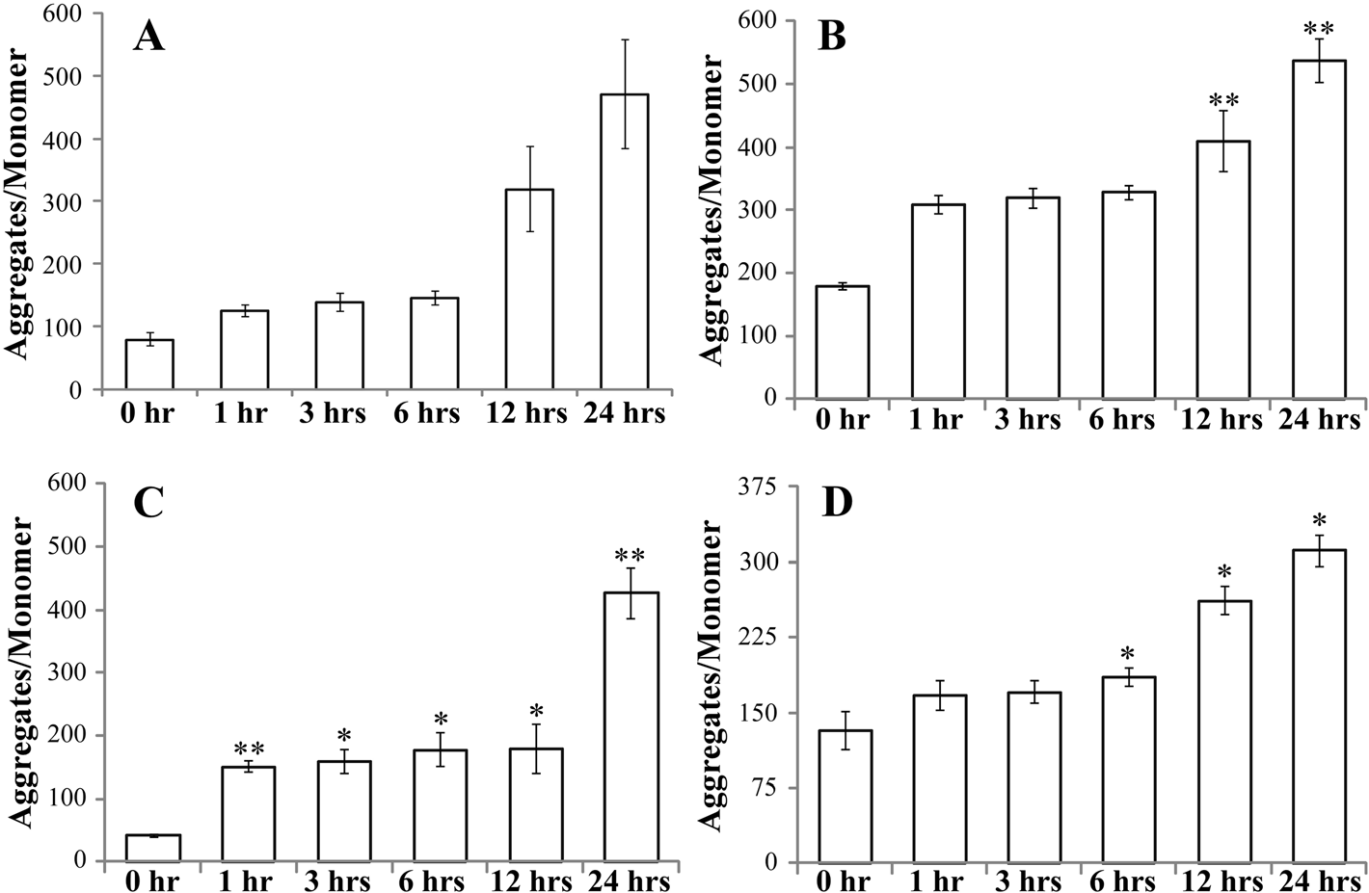
The effect of CycT treatment on mitochondrial membrane potential in NSCLC H1299 (**a**), A549 (**b**), and H460 (**c**) cells. **d** SANT1 treatment also increases mitochondrial membrane potential in NSCLC H1299 cells. NSCLC cells were treated with CycT or SANT1 for the indicated time periods. Then mitochondrial membrane potential in these cells was measured by using JC-1 staining. Mitochondrial membrane potential was expressed as the ratio of aggregates/monomer, which was calculated by dividing red fluorescence intensity with green fluorescence intensity. For statistical analysis, the levels in CycT-treated cells were compared to the levels in untreated cells, by using Welch 2-sample *t*-test. *, *p* value < 0.05; **, *p* value < 0.005

To further investigate the mode by which CycT causes NSCLC cell death, we measured ROS generation in CycT-treated and untreated NSCLC cells. We found that CycT causes a substantial increase in ROS generation in NSCLC cells, including H1299 (Fig. 4a), A549 (Fig. 4b), and H460 (Fig. 4c) cells. In addition, we found that another SMO inhibitor SANT1 increased ROS generation in NSCLC cells (Fig. 4d). Because of the dominant role of mitochondria in oxygen metabolism, mitochondria are mainly responsible for the generation of cellular ROS. Therefore, we also examined the effect of CycT on mitochondrial function. First, we measured and compared mitochondrial membrane potential in CycT-treated and CycT-untreated cells. We found that CycT increases mitochondrial membrane potential substantially in NSCLC cells (Fig. 5a, b and c). This effect of CycT appeared to be stronger in H1299 (Fig. 5a) and H460 (Fig. 5c) cells than in A549 cells (Fig. 5b). Likewise, another SMO inhibitor SANT1 increased mitochondrial membrane potential in NSCLC cells (Fig. 5d).

### CycT causes mitochondrial fragmentation in NSCLC cells

**Fig. 6:**
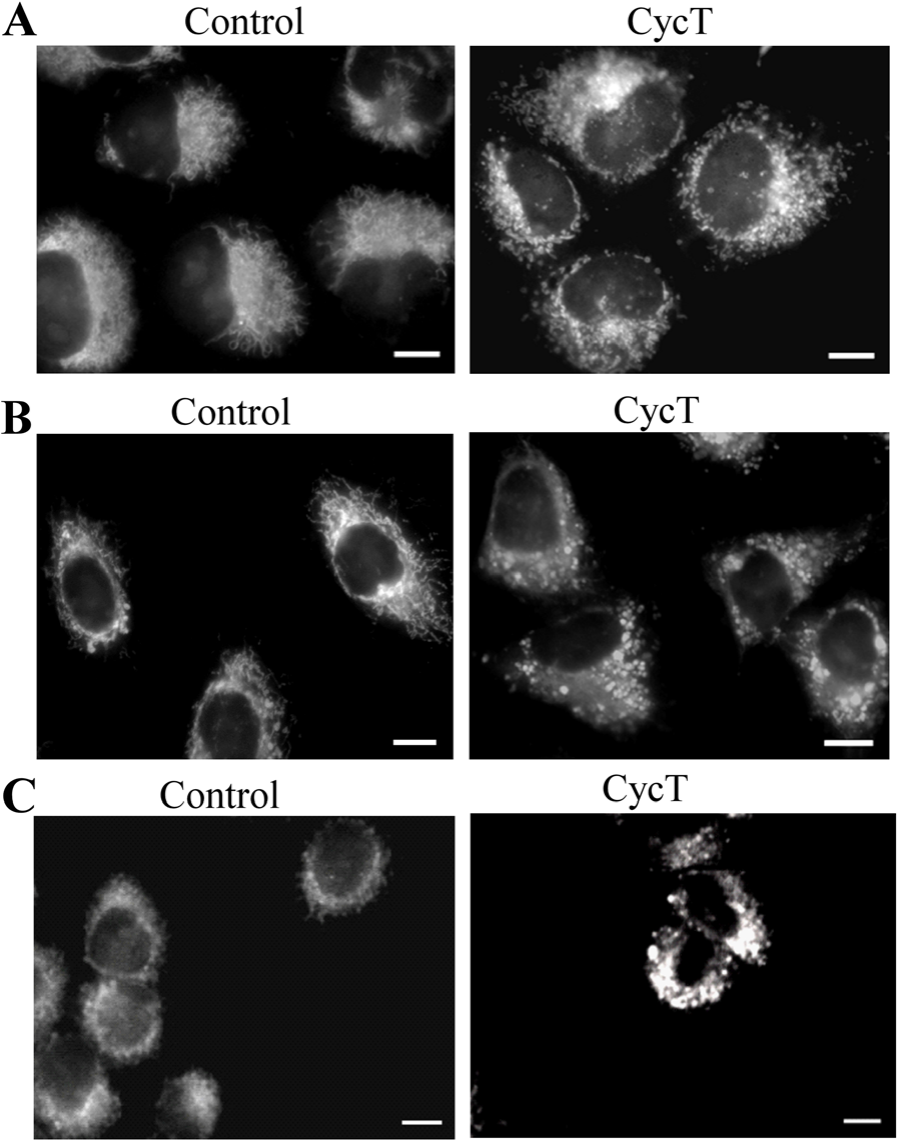
CycT treatment causes mitochondrial fragmentation in NSCLC H1299 (**a**), A549 (**b**), and H460 (**c**) cells. NSCLC cells were treated with CycT for 24 h, and then stained with MitoTracker Red. Fluorescent images were acquired and shown here. The scale bar indicates 10 μm

Mitochondrial morphology is closely linked to mitochondrial function [25]. Changes in mitochondrial morphology regulate many mitochondrial functions, such as the respiratory activity of the electron transport chain and apoptosis [26]. We therefore examined the effect of CycT on mitochondrial morphology in NSCLC cells by using MitoTracker Red. Fig. 6 shows that CycT treatment caused mitochondrial fragmentation in NSCLC cells, H1299 (Fig. 6a), A549 (Fig. 6b), and H460 (Fig. 6c) cells. SANT1 exerted similar effects on the NSCLC cells (not shown). Because Drp1 is the main protein promoting mitochondrial fission and fragmentation [27], we also examined the effect of CycT on Drp1 distribution. Additional file 2: Fig. S2 shows that in CycT-treated NSCLC H1299 (Additional file 2: Fig. S2A) and A549 (Additional file 2: Fig. S2B) cells, Drp1 were indeed selectively localized to various mitochondrial fission sites: The localization pattern of Drp1 completely coincided with the MitoTracker Red staining pattern. These results demonstrated that CycT can induce Drp1 to promote mitochondrial fission and fragmentation.

## Discussion

Targeting Hh signaling is an important strategy that is being developed to treat a variety of metastatic and advanced cancers [9, 28]. Vismodegib, a SMO antagonist, was the first Hh inhibitor to receive approval from the USA FDA in January 2012 for the treatment of locally advanced or metastatic basal cell carcinoma (BCC) [29]. Currently, NIH lists 91 ongoing or completed clinical trials testing Hh inhibitors against a variety of cancers (https://clinicaltrials.gov/ct2/results?term=HEDGEHOG). Therefore, a comprehensive understanding of the modes of Hh inhibitor action can benefit the treatment of a wide range of cancers. Here, by using the well-studied Hh inhibitor cyclopamine tartrate, we identified a new activity of this drug against aerobic respiration and mitochondrial function in NSCLC cancer cells.

First, we found that oxygen consumption is intensified in an array of NSCLC cell lines and that glutamine has a prominent role in promoting oxygen consumption, because glucose depletion in the presence of glutamine enhanced oxygen consumption while glutamine depletion in the presence of glucose diminished oxygen consumption substantially (Fig. 1). This is entirely consistent with previous studies showing glutamine as the dominant respiratory substrate in tumor cells [30, 31]. Remarkably, we found that CycT exerted largely the same effect on oxygen consumption as glutamine depletion (see Fig. 1b-g). Glutamine provides a crucial source of cellular energy and building blocks for cancer cells, and targeting glutamine uptake and metabolism has become an important approach in treating drug-resistant cancers [32, 33]. Our finding linking both CycT treatment and glutamine depletion with diminished aerobic respiration provides a new way to control cancer cell bioenergetics. Although different NSCLC cell lines exhibited varying rates of oxygen consumption (Figs. 1b-g), CycT, like glutamine depletion, strongly diminished oxygen consumption in all cell lines, suggesting that CycT can be an effective inhibitor of cellular energy production in all these lines. The same effect of glutamine depletion and CycT treatment suggests that Hh inhibitors may be used in substitution of drugs targeting glutamine metabolism in cancer therapy.

Second, we showed that CycT strongly suppressed NSCLC cell proliferation (Additional file 1: Fig. S1) and induced apoptosis (Fig. 2a and b). Notably, apoptosis of NSCLC cells was preceded by increased ROS generation (Fig. 4a and b) and increased mitochondrial membrane potential (Fig. 5a and b). At first sight, increased mitochondrial membrane potential accompanying apoptosis may seem paradoxical. However, hyperpolarization of mitochondrial membrane potential induced by various stress factors have been observed in a wide array of cells ranging from neuronal to blood cells [34]. For example, both glucose and oxygen deprivation induces mitochondrial membrane potential hyperpolarization [35]. Hyperpolarization of mitochondrial membrane potential will lead to excessive ROS production [34, 36]. The connection between the ΔΨm and ROS is exponential when ΔΨm exceeds 140 mV [37]. Ultimately, increased ROS generation will lead to apoptosis [38, 39], as indicated in Figs. 4 and 5.

Third, we found that CycT strongly induced mitochondrial fission and fragmentation (Fig. 6). As expected, CycT-induced mitochondrial fragmentation accompanied the recruitment of Drp1 to the mitochondrial outer membrane and fission sites (see Additional file 2: Fig. S2), consistent with previous models of mitochondrial fission [27]. Mitochondrial fragmentation is often associated with apoptosis, although it can occur in a variety of conditions independently of apoptosis [25, 40]. Here our results suggest that CycT-induced apoptosis in NSCLC cells is associated with mitochondrial fragmentation. Interestingly, while CycT caused strong mitochondrial fragmentation in both A549 and H1299 cells, apoptosis was much less prominent in A549 cells (Fig. 2), suggesting differential sensitivity of NSCLC cells to mitochondrial fragmentation and CycT.

In sum, our results provide novel insights into the potential mechanisms of action of Hh signaling pathway inhibitors. Previous studies have shown that Gli transcriptional factors are the key mediators of Hh signaling [1, 2]. Furthermore, genome-wide analyses using gene expression profiling and chromatin immunoprecipitation have identified many Gli target genes [41–43]. A careful examination of these target genes showed that there are 30 genes encoding for mitochondrial functions, such as MRPL23, GLUL, SLC25A13, PRDX6, and ATP6V1E1 [41]. These targets also include NDUFS8, a subunit of mitochondrial NADH:ubiquinone oxidoreductase, or Complex I; Cyb5b, a cytochrome b5 outer mitochondrial membrane isoform; 1810063B05Rik, cytochrome c oxidase assembly factor 6; and catalase. Although the identification of such a relatively small number of mitochondrial targets does not allow the inference of a global effect of Hh signaling or Hh inhibitors on mitochondria, it does lend support to our new finding that the Hh inhibitor CycT impacts mitochondrial morphology and function, thereby modulating mitochondrial respiration and apoptosis. Further studies comparing SMO inhibitors, such as CycT, and Gli inhibitors may provide additional insights into which proteins in the Hh signaling pathway play a dominant role in mitochondrial morphology and function.

## Conclusion

The major findings of our study are: a) CycT, like glutamine depletion, causes a substantial decrease in oxygen consumption in a number of NSCLC cell lines; b) CycT suppresses proliferation and induces apoptosis in NSCLC cells; c) CycT and evidently other Hh inhibitors promote mitochondrial fission and fragmentation, mitochondrial membrane hyperpolarization, and ROS generation; and d) Hh signaling inhibitors can act on mitochondria and cause broad and dramatic changes in mitochondrial morphology, respiration and function. These new findings can shed light on the mechanisms underlying various cancers associated with aberrant Hh signaling and can provide novel insights into how to optimize anti-Hh signaling strategy for treating cancer.

## Methods

### Lung cell lines, antibodies and reagents

HBEC30KT and HCC4017 cell lines representing normal and NSCLC cells [44, 45] were provided by Dr. John Minna’s lab (UTSW) as a gift. They were developed from the same patient and were maintained in ACL4 supplemented with 2 % FBS under 5 % CO_2_ at 37 °C [45]. All other NSCLC cell lines, H1395, H1299, Calu-3, A549 and H460 were purchased from ATCC, and were maintained according to ATCC procedures. All tissue culture media, including those lacking glucose or glutamine, were purchased from Invitrogen Life Technologies. HBEC30KT, HCC4017, H1299 and A549, were authenticated by using the services provided by Genetica DNA Laboratories, and they were found to be 100 % match. Other NSCLC cell lines were used for measurements immediately following the purchase. Cyclopamine tartrate (CycT) was provided by Logan Natural Products (Plano, Texas). SANT1 was purchased from Santa Cruz Biotechnology. For measuring the effect of CycT or SANT1 on lung cell proliferation, cells were seeded in 48-well plate at a density of 10^4^ cells/well. After culturing for 24 h, cells were treated with the indicated concentrations for 24 h or the indicated time points. At the indicated times, the number of live cells was counted by using trypan blue staining and a hemocytometer. Polyclonal anti-ALAS1, anti-HO1, anti-Gli1 and anti-Drp1 were purchased from Santa Cruz Biotechnology. Monoclonal anti-vinculin antibody was purchased from Sigma-Aldrich. Polyclonal anti-phospho-p44/42 MAPK antibody was purchased from Cell Signaling Technology.

### Measurement of oxygen consumption rates

NSCLC cells (∼70 % confluence) were maintained in medium with 25 μM CycT or 50 μM SANT1, or in medium lacking glucose or glutamine for 24 h. Then oxygen consumption was measured, as described previously [46]. Briefly, cells with about 80 % confluence were trypsinized and resuspended in fresh, air-saturated medium. For each measurement, 10^6^ cells (in 350 μl) were introduced in the chamber of an Oxygraph system (Hansatech Instruments), with a Clark-type electrode placed at the bottom of the respiratory chamber. During measurements, the chamber was thermostated at 37 °C by a circulating water bath. An electromagnetic stirrer bar was used to mix the contents of the chamber. Each measurement was replicated at least three times. Standard deviations were calculated, and p values were calculated by using Welch 2-sample *t*-test (R program).

### Measurement of ROS generation and mitochondrial membrane potential

The intracellular ROS levels were measured by using 2,7-dichlorodihydrofluorescein diacetate (DCFH-DA) (Cayman Chemical), which is oxidized into highly fluorescent 2,7- dichlorofluorescein in the presence of intracellular ROS. Cells were seeded in 96-well plates with black walls and clear bottoms at a density of 10,000 cells per well. Cells were treated with 25 μM CycT or 50 μM SANT1 for the indicated times. Cells were then incubated for 30 min with 10 μM DCFH-DA dissolved in fresh media. Cells were then rinsed twice with PBS, and each well was filled with 100 μl PBS. Fluorescence was detected by using a fluorescent plate reader (Bio Tek, Synergy Mx microplate reader) with the excitation and emission wavelengths at 490 and 535 nm, respectively.

Changes in the mitochondrial membrane potential (ΔΨm) were measured quantitatively by staining with the cationic dye JC-1(Molecular probes), which accumulates in the mitochondria, showing green fluorescence at lower membrane potential and forming J-aggregates with red fluorescence at higher membrane potential. Cells were seeded in 96 well black wall and clear bottom plates at a density of 10,000 cells per well. Cells were treated with 25 μM CycT or 50 μM SANT1 for the indicated times. Cells were then incubated with 200 μl of fresh medium containing 2 μg/μl of JC-1 dye for 30 min in the dark. The cells were washed twice with PBS, and the plates were immediately read with a fluorescent plate reader (Bio Tek, Synergy Mx microplate reader) with excitation and emission wavelengths set at 540 and 595 nm, respectively, for red fluorescence; and 485 and 535 nm, respectively, for green fluorescence.

### Preparation of protein extracts and Western blotting

NSCLC cells were treated, collected, and lysed by using the RIPA buffer (Cell Signaling Technology) containing the protease inhibitor cocktail. Protein concentrations were determined by using the BCA assay kit (Thermo Scientific). 50 μg of proteins from each treatment condition were electrophoresed on 9 % SDS–Polyacrylamide gels, and then transferred onto the Immuno-Blot PVDF Membrane (Bio-Rad). The membranes were probed with polyclonal antibodies, followed by detection with a chemiluminescence Western blotting kit (Roche Diagnostics). The signals were detected by using a Carestream image station 4000MM Pro, and quantitation was performed by using the Carestream molecular imaging software version 5.0.5.30 (Carestream Health, Inc.). Antibodies used were purchased from Santa Cruz Biotechnology and Sigma-Aldrich.

### Mitochondria imaging and indirect immunofluorescence staining

Mitochondria were visualized by using Mito Tracker Red CMXRos (Molecular probes), which passively diffuses across the plasma membrane and accumulates in active mitochondria. Cells were grown on chamber slides and treated with CycT for 24 h. Cells were then stained with 200 nM of CMXRos in complete growth medium for 30 min at 37 °C, washed with prewarmed PBS three times, and fixed with 4 % formaldehyde in PBS for 10 min. After washing twice with PBS the slides were covered, and fluorescent images were captured with a multi-channel Zeiss Axio Observer Z1 fluorescent microscope with a Zeiss 40X Oil immersion lens and with a high speed AxioCam MRm Rev3 monochrome camera.

Indirect immunofluorescence staining with Drp1 antibodies (purchased from Santa Cruz Biotechnology) was performed by following the procedures provided by the antibody manufacturer. FITC and DAPI fluorescent images were captured by using a multi-channel Zeiss Axio Observer Z1 fluorescent microscope. Apoptosis was detected by using the ApoAlert Annexin V-FITC Apoptosis Kit (Clontech). Cells were seeded in a 96-well black wall clear bottom plate at the density of 10,000 cells per well. After one day, cells were treated with 25 μM CycT or SANT1 in fresh medium. Twenty four hours post treatment, apoptosis assay was performed according to manufacturer’s protocol. Fluorescent images were captured using a multi-channel Zeiss Axio Observer Z1 fluorescent microscope with a Zeiss 20X lens and with a high speed AxioCam MRm Rev3 monochrome camera.

### Ethics approval and consent to participate

There was not any ethics approval or consent required for the use of human derived cell lines in this study.

## Additional files

Additional file 1: Figure S1. The effect of CycT treatment on NSCLC cancer cell proliferation. %live cells was calculated by dividing the number of treated cells with the number of untreated cells (seeded with the same number of cells). It shows the relative proliferative rates of treated cells (10 or 25 μM) vs. untreated cells (None). For statistical analysis, the values for treated cells were compared to the values for untreated cells, by using Welch 2-sample *t*-test. *, *p* value < 0.05; **, *p* value < 0.005. (JPG 270 kb) Additional file 2: Figure S2. Drp1 localizes to the mitochondrial fission sites in CycT-treated NSCLC H1299 (A) and A549 (B) cells. NSCLC cells were treated with CycT for 24 h. Cells were incubated with anti-Drp1 antibodies, and then with FITC-conjugated goat anti-rabbit secondary antibody, MitoTracker, as well as DAPI. FITC, MitoTracker and DAPI fluorescent images were captured and are shown here. The scale bar indicates 10 μm. (TIF 15003 kb)

## Abbreviations

ALAS1: 5’-Aminolevulinate Synthase 1
BCC: basal cell carcinoma
CycT: cyclopamine tartrate
DAPI: 4′, 6-Diamidino-2-phenylindole
DCFH-DA: 2,7-dichlorodihydrofluorescein diacetate
GLUL: glutamate-ammonia ligase
HO1: heme oxygenase 1
MRPL23: mitochondrial ribosomal protein L23
NDUFS8: NADH Dehydrogenase (Ubiquinone) Fe-S Protein 8
NSCLC: non-small-cell lung cancer
PRDX6: peroxiredoxin 6
ROS: reactive oxygen species
