# Supplementary figures and images for: Cyclopamine tartrate, an inhibitor of Hedgehog signaling, strongly interferes with mitochondrial function and suppresses aerobic respiration in lung cancer cells
Source: BMC Cancer. 2016 Feb 24;16:150. doi: 10.1186/s12885-016-2200-x (PMC4766751; doi:10.1186/s12885-016-2200-x)

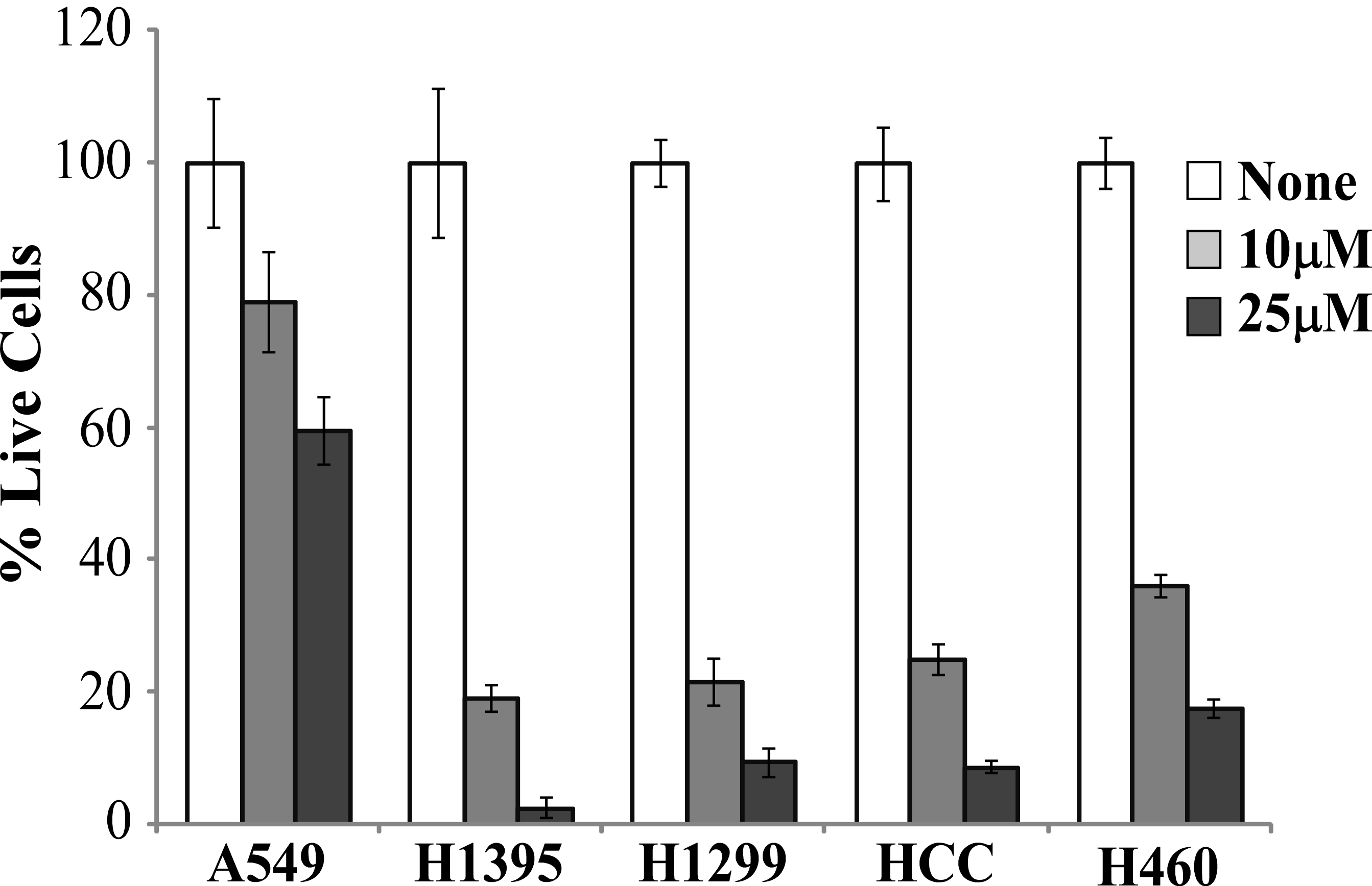

Supplement: Additional file 1: Figure S1. — The effect of CycT treatment on NSCLC cancer cell proliferation. %live cells was calculated by dividing the number of treated cells with the number of untreated cells (seeded with the same number of cells). It shows the relative proliferative rates of treated cells (10 or 25 μM) vs. untreated cells (None). For statistical analysis, the values for treated cells were compared to the values for untreated cells, by using Welch 2-sample t-test. *, p value < 0.05; **, p value < 0.005. (JPG 270 kb) [file 12885_2016_2200_MOESM1_ESM.jpg]

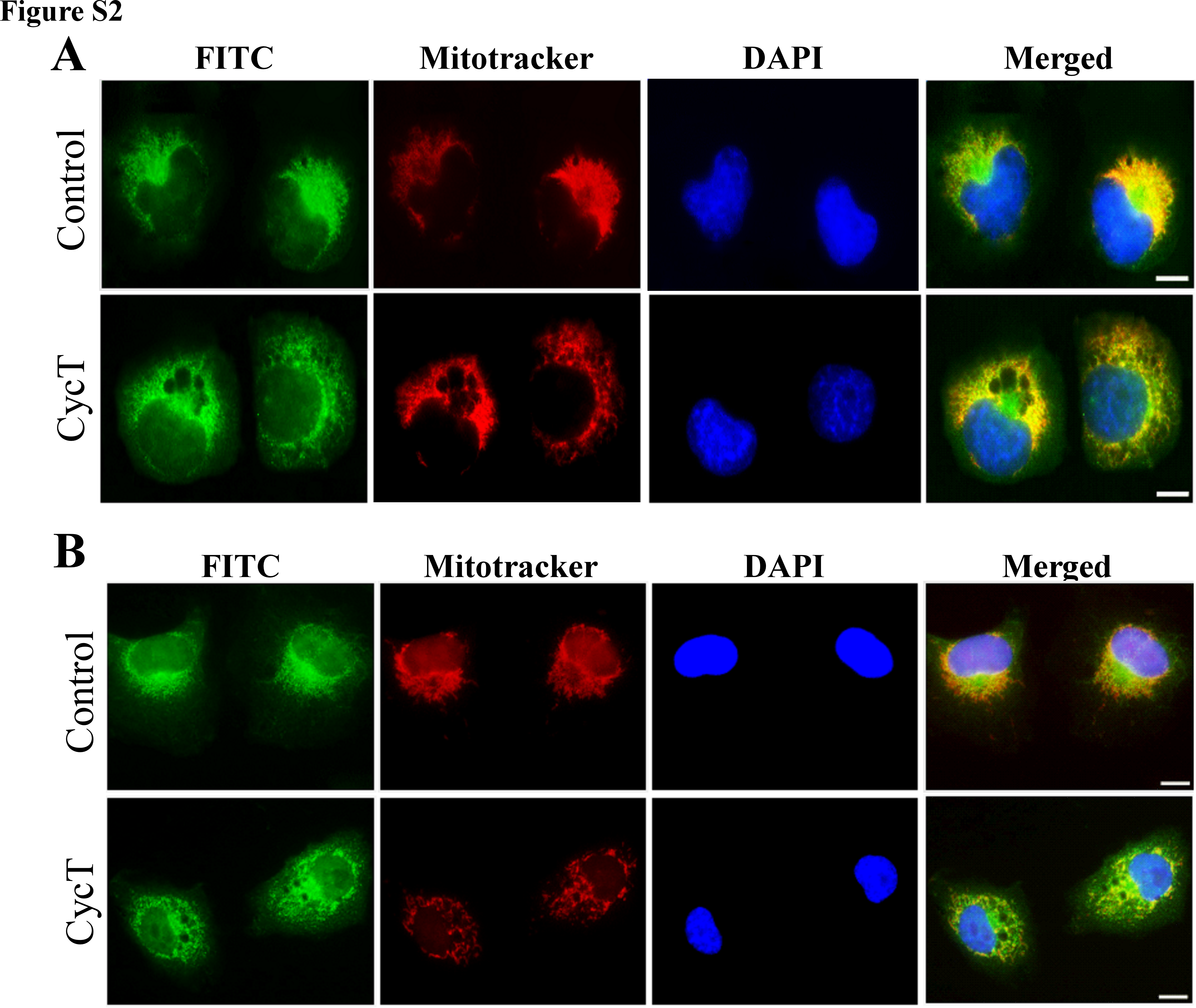

Supplement: Additional file 2: Figure S2. — Drp1 localizes to the mitochondrial fission sites in CycT-treated NSCLC H1299 (A) and A549 (B) cells. NSCLC cells were treated with CycT for 24 h. Cells were incubated with anti-Drp1 antibodies, and then with FITC-conjugated goat anti-rabbit secondary antibody, MitoTracker, as well as DAPI. FITC, MitoTracker and DAPI fluorescent images were captured and are shown here. The scale bar indicates 10 μm. (TIF 15003 kb) [file 12885_2016_2200_MOESM2_ESM.tif]
